# Feasibility of Adding Supplemental Solid Rubber Mats to a Confined Slatted Barn Cattle Feedlot System

**DOI:** 10.3390/ani15202978

**Published:** 2025-10-14

**Authors:** Courtney A. Hayes, Jackson B. Matthews, Benjamin W. Blair, Jonathan H. Foreman

**Affiliations:** 1 Department of Veterinary Clinical Medicine, College of Veterinary Medicine, University of Illinois Urbana-Champaign, Urbana, IL 61802, USA; bblair2@illinois.edu (B.W.B.); jhf@illinois.edu (J.H.F.); 2 Department of Animal Sciences, College of ACES, University of Illinois Urbana-Champaign, Urbana, IL 61802, USA; jbm3@illinois.edu

**Keywords:** feedlot cattle, lameness, supplemental mats, cleanliness scoring, lameness scoring, welfare

## Abstract

**Simple Summary:**

Lameness is known to impact many feedlot cattle and is both a welfare and a management concern. For cattle housed indoors, the quality of the lying surface may be a contributing factor. Early-weaned beef cattle entering indoor feedlot facilities in the winter were provided supplemental rubber mats for three months. This management change was implemented to determine if creating a more comfortable lying area was feasible and if the animals would cope well with this flooring change. Additionally clinical, behavioral, and other welfare variables were observed to help determine normal behavior of these calves and if the mats were causing any problems. Both heifer and steer calves were used in this project, and differences were observed in their outcomes.

**Abstract:**

Indoor housed cattle, particularly those housed in slatted floor barns, may develop specific types of lameness associated with their housing environment. Previous studies have demonstrated that cattle raised on slats that are fitted with rubber perform better than cattle that are on concrete slats alone; however, lameness continues to be a problem even with this modification. This project investigated the feasibility of adding additional commercially available solid mats to the rubber-coated slatted floor barn and observing animal behavior and outcomes in a group setting. The objective was to determine if creating an improved lying area through a relatively simple management change could positively impact the outcome of these animals. Commercial mats were simple to install and were used immediately and extensively by the cattle. However, the outcome provided mixed results. The additional mats provided challenges with cleanliness. Steer calves became dirty faster and more severely than heifers. Forty-three percent of the heifer calves and 19% of the steers were culled early. More work is needed to better understand and provide solutions for this welfare issue.

## 1. Introduction

Barns with slatted floors have been used in Europe and North America to raise livestock since the 1960s [1,2]. Intensification of production as well as a focus on efficiency are some of the reasons for their development and use. A slatted floor barn allows urine and feces to fall through gaps in the floor into a collection area or pit, separating waste from the animals. Conventionally, for cattle and pigs, the animal flooring is comprised of concrete “slats” that are of a uniform size placed parallel to each other with a specific size gap in between [3]; however, other configurations or materials such as fiberglass or expanded metal [2], wood [4], or plastic [5] may be used for the slats depending on the production class of animal. Cattle [1], pigs [2], sheep [4], and poultry [5] can all be raised in slatted floor barns.

One of the advantages, from a management standpoint, of a slatted floor system is the elimination of the use of bedding [6]. From the producer’s perspective, operating an animal facility without bedding saves money on both bedding material and labor. It avoids dependence on straw, shavings, or paper byproducts, items subject to market fluctuations, weather fluctuations, and industry production. Slatted floor systems also reduce labor costs, because there is no need for cleaning and re-bedding while animals are in the barn [7]. However, for the animals, a lack of bedding may impact their lying and standing behavior and reduce confidence in their footing [8].

One of the welfare benefits to cattle fed indoors on slats is protection from detrimental weather conditions such as freezing rain and extreme heat. There are also management benefits that coincide with health benefits to the animals, such as feed being protected from the elements, regular monitoring of health status, and easy access to animals for medical treatment [9]. The lack of bedding also creates a reduction in external parasite infestation.

Slatted floor barns are designed to be more environmentally friendly than outdoor feedlots. They retain manure nutrients, reduce concerns about ground water contamination, and reduce odors [10]. They are designed to be used without bedding [3]. Because of this design, bedding cannot be used, because it would fall through the slats and impact the ability to remove manure from the pit; therefore, adding bedding to improve welfare is not an option without a significant re-design of the barns.

The European Union (EU) uses slatted floor barns to house cattle of various life and production stages. These barns are frequently used as short-term winter housing for dairy cattle, calves, and bulls [11]. The EU finishes 9.1 million animals per year in feedlots [11]. Indoor slatted floor systems are the most common feeding systems in Europe [12]. Common breeds found in these feeding systems include Limousin, Charolais, Chianina, and Belgian Blue, also known as continental breeds. These breeds are typically slower to mature and reach a heavier finishing size than many of the feedlot animals in the United States (US).

In the US there are currently an estimated 14.4 million cattle in feedlots. The United States Department of Agriculture (USDA) categorizes feedlots as “large” if they house 1000 animals or more and “small” if they house between 50 and 999 animals [13,14]. The USDA uses cash receipts for agricultural commodities to classify most of these operations [15]; feedlots smaller than 50 head may not be identified in this manner, as they tend to source the animals from their own cow-calf herds. Small feedlots contribute to the US industry with 2.49 million head of cattle on feed. While most feedlot cattle in the United States are fed to market weight in extensive outdoor systems, many smaller feedlots, particularly in the Upper Midwest and Eastern states, utilize intensive indoor systems. These facilities vary in design, typically providing shelter from inclement wet weather, protection from heat stress, and improvement in manure management in areas with high rainfall, which are common problems for outdoor feedlots [16]. There are several different types of flooring in these indoor facilities including either partial or fully slatted floors.

There is no national census for animals on feed by facility type, but a 2014 survey [10] indicated that, of cattle finished in Iowa, 4% of the animals were in deep-pit confinement buildings (slatted floor barns). Iowa is one of the top five cattle producing states, with 1.18 million head on feed. Smaller cattle-producing states such as Michigan have reported 42% of their feedlots are slatted floor barns [17]. Michigan currently has 135,000 cattle on feed [18]. Other Midwestern states such as Illinois have 250,000 cattle on feed [19], while Northeastern states such as Pennsylvania have 211,000 cattle on feed [20]. It would be reasonable to estimate that 45,000–50,000 animals in each of the cattle-producing states in these regions of the US are impacted by being raised in this type of facility every year.

Historically, respiratory disease has been considered the primary health and welfare concern for feedlot cattle [21]. However, this concern is primarily based on data from large outdoor feedlot systems. Smaller feedlots often mitigate respiratory disease risks due to fewer cattle and localized purchasing, reducing the need for extensive transportation and minimizing transport stress and the commingling of animals, which are significant contributors to respiratory disease [22]. Additionally, metaphylactic antimicrobial therapy is a common and legal practice in North American feedlots [23]. Finally, knowledge and research about the risk factors associated with bovine respiratory disease (BRD) have changed management practices and have positively impacted feedlot cattle in this regard. In smaller indoor systems, lameness may pose a greater welfare challenge.

Lameness is a known welfare issue in finishing cattle in both the US and the EU [7,24]. It is known to be a painful condition and can lead to early culling and even euthanasia. Delayed detection of this painful condition is of particular concern. Lameness is a multi-factorial disease, and the severity and incidence of this disease process in indoor housed cattle is not well-defined in the literature. Cattle can suffer from infectious etiologies, such as foot rot and digital dermatitis [25], or mechanical injuries, such as claw deformities [26,27] and stress fractures [28]. Complicating diagnosis, common clinical signs in lame animals such as joint swellings can be the result of either infection [29] or injury [30]. Risk factors for different forms of lameness depend on the management system. Cattle housed on slatted floors are at lower risk for conditions like foot rot and toe abscesses due to reduced exposure to mud and manure, but they may still suffer from mechanical injuries due to inadequate flooring. Therefore, it is crucial to differentiate between the risk factors associated with lameness in indoor and outdoor systems.

The dairy industry has a significant body of work documenting causes of lameness for cows housed on concrete, as well as methods of improving the facilities to reduce the associated lameness. Such improvements include the addition of rubber coverings on walking surfaces [31], and the provision of a comfortable lying area. Cow preference tests and time budget analyses of cow activity have increased understanding of the needs of Holstein cows [32,33,34]. However, this information is not yet available to the same degree for dairy youngstock or beef animals. One of the welfare concerns with the use of indoor slatted floor housing is that finishing cattle are not provided a comfortable lying area [35].

Smaller space allowances and abrasive flooring surfaces are suspected to contribute to a higher lameness incidence in indoor systems compared to large outdoor systems [36]. Previous studies [37,38] have shown an improvement in welfare outcomes when rubber mats are fitted to the slats, in comparison to bare concrete slats. Slats covered with fitted rubber mats to improve footing are commonly used now, with newer facilities being built and older ones being retrofitted with mats. Most producers in the US currently use rubber matting in their slatted floor barns [39]. However, lameness continues to be an issue even in barns with rubber-covered slatted floors.

Many factors can impact the level and degree of lameness experienced by these animals, including breed [40], conformation, stocking density, and length of days [36,39] on slats. The longer the animals are on slats, the higher the incidence of lameness. Dawson et al. [39] reported that, for cattle housed on slatted floors, gait and mobility worsened with a greater numbers of days on feed regardless of floor type: bare concrete slats, slats covered with new rubber mats, or slats covered with aged rubber mats. Elmore et al. [36] also found that gait scores worsened over time regardless of treatment. Although most traditionally raised beef calves in the US continue to be weaned at 6–8 months of age, dairy calves raised for beef, including the boom of dairy beef crosses, can potentially enter a feedlot at much younger ages and be housed on slats for longer periods of time. Because the US Eastern region and Upper Midwest are both more likely to use these types of indoor barns, as well as to raise dairy and dairy-crossed beef [41], it is reasonable to expect earlier weaned calves to be housed on slats in these regions of the country. Financial factors such as the availability of calves, calf price, and open space in a barn may encourage farmers to place calves in confinement rearing facilities at earlier ages.

There are a number of scientific studies [37,38,42] that have investigated the effects of different types of flooring on welfare and production in growing beef animals. Many of these studies occurred in Europe, where beef animals are weaned and enter the feedlots at a later age than in the US. None address early-weaned calves entering the feedlot and the incidence of lameness in this population of animals. Additionally, due to the experimental nature of these projects, animal numbers in groups are small, usually 2–7 per pen. Practical measures to assess lameness in commercial groups of cattle have also not been established.

The purposes of this project were to observe the normal behavior of newly arrived calves housed indoors in a slatted barn, monitor for signs of developing lameness, and to determine if adding a more comfortable lying area that did not involve bedding might improve their welfare. There is a gap in the literature with respect to what modifications to this type of barn would be most impactful on the prevention of lameness and at what age; however, one study from the US [36] demonstrated that finishing steers showed a preference for resting on solid rubber mats compared to bare concrete slats.

The lying area in this project was to be created with multiple straight-edged mats placed adjacent to each other on top of the already rubberized slats, resulting in a thicker area of rubber. The mats chosen were made of vulcanized rubber and characterized as firm yet resilient [43]. They had a textured surface, pebble-top, designed for non-slip traction.

The premise was that providing a section of solid rubber flooring on top of the rubber-coated slats would provide an area that protects from drafts coming up from the pit, provides a softer area to lie down, and potentially allows the animals to take longer strides. The goal of this project was, therefore, to determine if adding supplemental rubber mats would be feasible in our finishing facility. A secondary goal was to help identify criteria that would be useful for producers to identify signs of early lameness in a production setting.

## 2. Materials and Methods

### 2.1. Background and Limitations

The University of Illinois Beef Research Unit consists of three cow-calf herds and a feedlot. The feedlot is comprised of six indoor confinement buildings with slatted concrete floors covered with rubber mats. Typically, 400–500 early-weaned calves enter the feedlot in late winter/early spring, and another 250–300 traditionally-weaned calves enter in the late summer/early fall, depending, in part, on feed availability and research needs. Lameness has been detected in both weaned groups of calves and is a closely monitored issue. Animals on research are regularly weighed (every 28 days) and are frequently locomotion scored at the same time. Animals also enter the feedlot for production purposes alone, in which case, they rarely leave their pens. Animals not on research will get weighed but not on a regular basis; they also do not get locomotion scored.

One major limitation to the design of this project was the lack of a control group. Another research project occurring concurrently limited the number of animals available for use in this cohort. This limitation prevented having a negative control group, as well as having replication pens. It also prevented selection of the location of the pens. An option would have been to divide the groups into two pens of heifer calves and two pens with steer calves, allowing one control pen for each gender. However, we were reluctant to do that, because historically, creating smaller pens at our facility has led to the type of biomechanical injuries we were trying to prevent [39]. Further subdivision would also have created a limited number of animals in each group, experimental and control, given the mixed gender of the available calves. It was felt that two larger groups without a negative control in this initial project was a better design than four smaller groups of limited numbers.

### 2.2. Study Design

This study was conducted to evaluate the feasibility of adding supplemental rubber mats to rubber-coated, fully slatted floor barns and to assess their impact on the welfare of early-weaned calves. The study was approved under the University of Illinois Institutional Animal Care and Use Committee (IACUC) Protocol #22163. Fifty-six calves (30 heifers and 26 steers) were enrolled in the study and housed in two adjacent pens with similar dimensions and space allowances. All animals were raised and owned by the University of Illinois; no private animals were procured.

### 2.3. Animals and Housing

Fifty-six Angus-Simmental calves were sourced from a fall-calving herd and arrived at the research facility in February, having been weaned in December and transitioned to solid feed. The calves ranged from 62 to 107 days of age at weaning, with an average age of 86 days. They remained at the home farm for 45 days, adjusting to solid feed prior to shipment to the feedlot. Upon arrival, calves were weighed and administered a single dose of a prophylactic antibiotic (Macrosyn^®^ Bimeda Inc., La Sueur, MN, USA). The calves were housed in a research barn measuring 68.9 × 12.8 m, with pens configured to simulate routine commercial feeding conditions. These calves were fed a corn silage-based total mixed ration once per day in the morning between 7 and 8 am Central Standard Time (shifted to the same feeding hour but in daylight savings time in March). There were 30 heifer calves housed in one pen, and 26 steer calves housed in a second. The animal numbers were predetermined by the source herd. The other three pens in the barn housed 23 steer calves per pen on a different project and were thus unavailable for use in our project.

**Figure 1:**
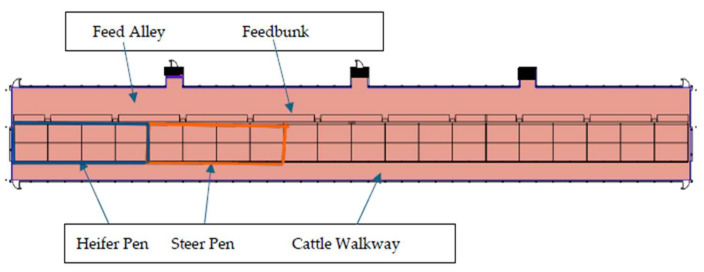
Barn layout with study pens; heifer pens outlined in blue and steers in orange.

The heifer calves were housed in a 4.9 × 19.5 m pen with 12.2 m of feedbunk space and two automatic waterers (Ritchie Industries Inc., Conrad, IA, USA). The adjacent pen housed the steer calves with the same dimensions and resources (Figure 1). This configuration provided 3.16 m^2^ of floor space per heifer calf and 3.6 m^2^ per steer calf, which is well above the minimum recommendations (1.1–1.7 m^2^) of the 2020 Ag Guide [44] and allometric calculations based on body weight [45].

### 2.4. Flooring Modifications

**Figure 2:**
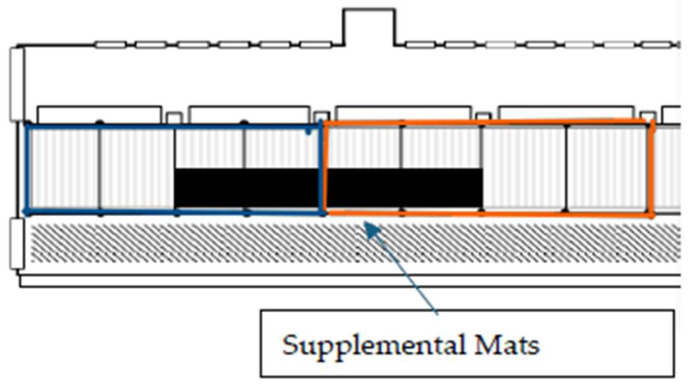
Pen layout with illustration of location of supplemental mats.

Both pens were equipped with fully slatted rubber-coated Animat Pebble mats (Animat, Sherbrooke, QC, Canada). To provide a cushioned lying area, sixteen 1.2 m × 1.8 m × 1.9 cm rubber stall mats (Flexgard^®^ stall mats, QRRI Inc., Sandy Springs, GA, USA) were purchased from a local farm supply store. Eight mats were placed in each pen on top of the existing rubber-coated slats, arranged side by side, covering a 1.8 × 9.8 m area, representing approximately 19% of the pen space (Figure 2). This provided an area where, in each pen, half of the back of the pen had supplemental mats and half did not. The mats selected were the largest horse stall mats available. The mats were not fixed in place and were located at the rear of the pens, away from the feedbunk, in the southeast corner of the heifer pen and the southwest corner of the steer pen (Figure 2).

### 2.5. Observations and Measurements

Calf behavior and welfare variables were monitored by a single observer (author CAH). The observer was a veterinarian with 25 years of cattle experience and 3 years of welfare-specific training. Initial observations were planned for a 30-day period, with three sessions per week, provided the mats remained in place and did not interfere with manure management. Because there was no movement of the mats and the calves were using them, the observation period was extended beyond the initial 30-day period with less frequent direct observations. The decision was made to leave the mats in for a total of three months. This time frame was chosen because it encompassed the period that the calves would be considered early-weaned.

Observations were conducted late in the afternoon three times per week during the first month and then weekly for the following month. Observation times as well as the ambient temperature at the time of observation were recorded. Most of the observations occurred around the same time of day, approximately nine hours after feeding. There were two observation points that were slightly earlier, closer to seven hours post-feeding. The times were chosen because they could be consistently relied upon to not have other human activity occurring in the barn, impacting calf behavior. This independent observation schedule was in addition to the twice-daily observations conducted by farm staff. Each observation period lasted 30 min and focused on the welfare domains [46] of environment and behavior. They included the following variables:

Ammonia concentrations: Measured at approximately the level of a calf’s head while lying down over both the mat and the slatted floor areas. This measurement was taken using a handheld ammonia meter (Forensics NH3000 Basic Ammonia Meter, Los Angeles, CA, USA) at the level of the 3rd bar from the ground of the walkway as far into the pen as could be reached. This location corresponded to a height of 68.5–69.9 cm and a distance inward of 40.6–43.2 cm.

Cleanliness: Visually assessed as a percentage of the group on a weekly basis. This was based upon the scale created by Elmore et al. in 2015 [36].

Score and description:

     Score of 1: <10% of body surface covered in manure.
     Score of 2: >10–25% of body surface covered in manure.
     Score of 3: >25–50% of body surface covered in manure.
     Score of 4: >50–75% of body surface covered in manure.
     Score of 5: >75% of body surface covered in manure.

Choice: Counting the number of calves lying on the mats versus on the slats. Counting the number of calves up at the feedbunk versus the ones simply standing inactive. Although oral manipulations were collected under play behaviors, animals performing these behaviors were included in the total count of animals as animals standing and performing an activity.

Physical changes or impacts: Recording the presence of knee and/or hock swelling and evidence of hair loss or pressure points on joints. Slips or falls when rising or moving about the pen were also recorded.

Play behavior: Recording the number of animals exhibiting any play behavior, including running, grooming, oral or head manipulation of gates and chains, and riding behavior.

The observations also included close inspection of the calves’ lower legs from the cattle walkway at the back of the pens (see Figure 1 diagram) to detect any signs of non-weight-bearing lameness or injury. The observer also noted any general changes in calf behavior in response to the presence of the observer.

Some of the individual behaviors observed but not quantified included eating and drinking. The two waterers in each pen were in line with the feeders, so it was not always possible to tell if the animals were at the feedbunk or at the waterer without getting close enough to disturb them. Additionally, once the animals noticed the presence of the observer, they often stopped what they were doing to watch the observer. Lying behavior observed included the location of the calves in the pen as well as their body and leg positions. The change of position from lying to standing was also observed and monitored for any signs of slipping or difficulty rising.

### 2.6. Health and Production Outcomes

Health outcomes, if applicable, including the incidence of lameness and other health problems, were recorded throughout the trial and through the feeding period. Additionally, animals that were removed beyond the period with supplemental mats but before the normal end of the feeding period were documented, along with their days on feed and the reasons for their removal.

Average daily gain (ADG) was calculated from weights collected during this period. Some comparative data on calves without access to supplemental mats were also collected for contextual analysis.

### 2.7. Locomotion Scoring

At the end of the trial, all calves were weighed, and their locomotion was scored as they exited the chute and moved across a dirt lot to a holding pen. Two independent observers used the 0–3-point Step-Up Locomotion Scoring System (Zinpro, Eden Prairie, MN, USA), which focuses on head movement, stride length, and detectable restricted limb use [39]. One observer was a veterinary technician with 15 years of cattle experience, and the other was a farm employee and graduate student in the field of Animal Science; both were trained in the use of the scoring system. Observers also noted any swollen joints or significant hair loss indicative of pressure points.

### 2.8. Tail Cleanliness Scoring

Also at the end of the trial, the cleanliness of the tails on all of the animals was assessed with a four score system progressing from “insignificant” to “mild” to “moderate” to “severe”. This new system was created based upon how much manure build up there was, as well as how difficult it was to remove manually. It was important to remove the manure, because many of the animals were carrying excess weight on their tails, and several had manure in a circumferential accumulation that could potentially have led to tail amputation.

### 2.9. Statistical Analysis

Average daily gain (ADG) was calculated for both heifers and steers. Data were analyzed using R Statistical Software (v4.1.2; R Core Team 2021) [47]. The data were evaluated for normal distribution using a Shapiro–Wilk normality test for each group. The data for each were normally distributed. Statistical comparisons between groups were made using a two-sample *t*-test, with significance set at *p* < 0.05. Although not an actual control group, data were available for ADG of steers without supplemental mats being fed the same diet. The data for this group were not normally distributed (the median was skewed to the right), so a Wilcoxon rank sum test was conducted to compare the distributions between this group of steers and the groups of steers on this study. For locomotion scoring, inter-observer agreement was assessed using Cohen’s kappa coefficient. Inter-observer agreement for the locomotion scores was high (ĸ = 0.94 for heifers and 0.82 for steers).

## 3. Results

At the beginning of the trial, the calves ranged in age from 117 to 163 days (average 144 days). By the end of the 89-day supplemental mat access period, calves averaged 235 ± 11 days of age.

**Figure 3:**
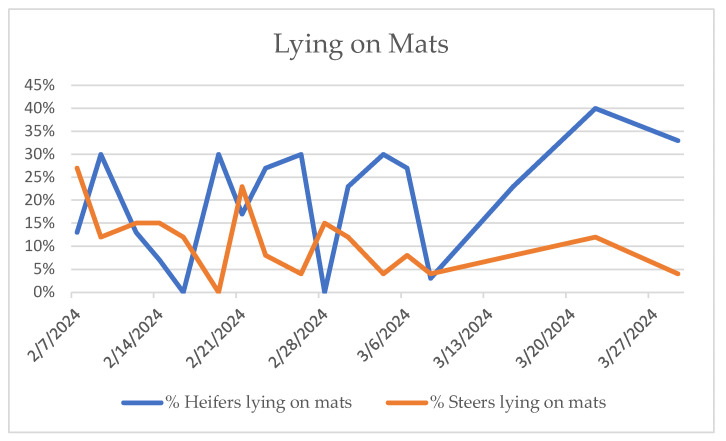
Percentage of animals observed lying on supplemental mats.

**Figure 4:**
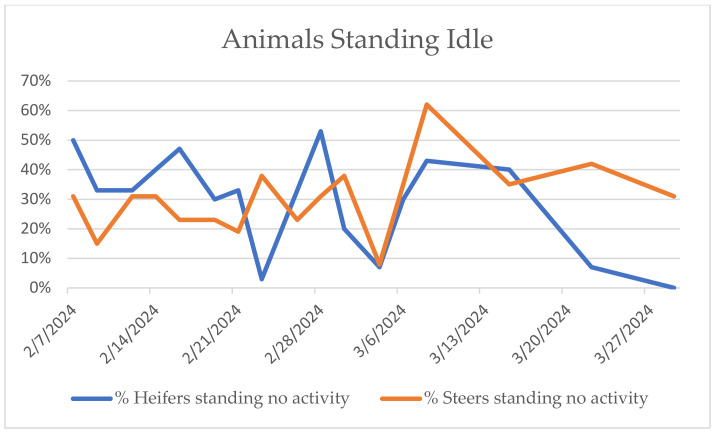
Percentage of animals standing with no activity.

Behavioral observations confirmed that calves used all pen areas for lying and standing, limiting conclusions from spatial behavior alone. At all observation times, some animals were at the feedbunk or the waterer, while the rest were lying down, standing, and manipulating gates or chains or just standing inactively. Resource guarding was not noted. Calves utilized the mats for both resting and standing. With the exception of the animals playing with chains and a few of those eating, calves noticed the presence of the observer very quickly. Calves would usually turn or walk towards the observer. On a couple of occasions, the calves ran towards the observer as a group. Calves lying down would immediately get up. Difficulties rising were not observed. As weeks went by and the calves became more used to the observer, the calves that were lying on the mats stopped getting up or would get up much later than the ones lying on slats. The calves that were standing on the mats would likewise remain standing on the mats rather than investigate the observer. Calves lying down were often in a sternal position with their legs tucked underneath them, although occasionally, there would be calves with a forelimb extended. Future studies that include a time budget of each of these activities would be informative to determine if more time, and if so, how much, was spent on the mats and in which positions. Figure 3 illustrates the percentage of animals lying on the supplemental mats at given time points, and Figure 4 illustrates the percentage of animals that were observed to be standing with no other activity. The steer calves used the mats for lying less over time.

**Figure 5:**
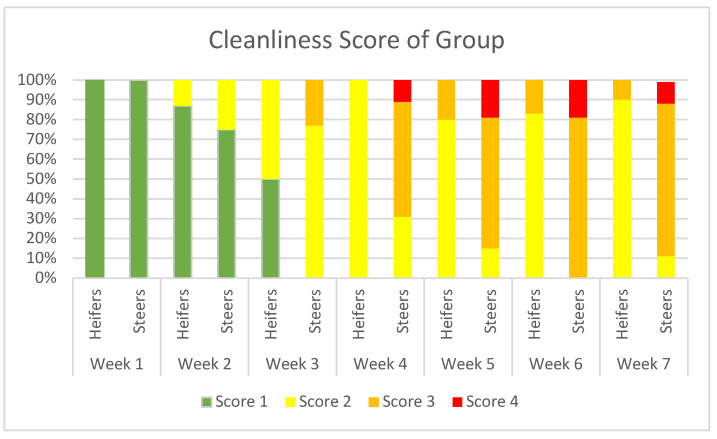
Progression of dirtiness over time.

Cleanliness was noted to be a concern early in the trial. This observation became more evident in the steer pen as time went by. Estimates of the percentage of animals with various areas of their body covered with manure were recorded (hoof to knee, abdomen, rear legs, rump, and tail). Due to the large group sizes, individual scoring was not possible. Up to 50% of the heifers were dirty up to the knees by the end of the first week. By the end of the first month, almost all the heifers received a score of 2 or 3, and all the steers received scores of 3 or 4 (see Figure 5). None of the animals observed became dirty enough to score a 5, and the peak of the dirtiness was observed at week 6 post-arrival, with a slight decrease recorded the following week. Over the last month on mats, the animals in both groups became larger, sleeker, and cleaner but never returned to a completely normal (Score 1) level of cleanliness.

No hair loss was observed. Joint swelling was observed in a few animals at the time they were locomotion scored but not prior to that.

Play behavior was limited. There was only one observation made of mutual grooming. No self-grooming was observed. There were three instances of riding and multiple occasions of one to three animals manipulating the gate chains or the gates themselves. Running occurred a few times when the animals in the pen noticed the observer. The animals always ran toward the observer.

Ammonia concentrations did not exceed 1 ppm at any measurement point for the two months ammonia was measured. There was no difference in measurements in the steer pen compared to the heifer pen.

There were a few isolated incidences of slipping noted; these all occurred when animals were moving and not when rising from lying down. No falls were seen. No animals were seen tripping on the edge of the mats.

No calves were removed from the study due to health reasons, including lameness, during the 89 days they had access to the supplemental mats.

Average daily gain (ADG) exceeded 1.8 kg of gain per day for the first 89 days, with the heifer calves gaining 1.88 ± 0.17 kg/d and the steers gaining 2.15 ± 0.21 kg/d. The ADG for the heifer calves was significantly less than that of the steer calves (*p* < 0.01). However, the ADG for the steers with access to supplemental mats was not significantly different from steers without mats housed in the same barn (2.15 ± 0.21 vs. 2.12 ± 0.20; *p* = 0.582). These data suggest that the additional mats did not have an effect on production.

Locomotion scoring was tallied in each category for total number of animals with that score (Table 1). Twenty-one heifer calves (70%) and 18 (69%) steer calves were given a score of zero (0) or normal. Only two animals in each group were given a score of two (2), 7% of heifer calves and 8% of steer calves, and no scores of three (3) were given. Seven heifer calves (23%) and three steer calves (12%) were identified by at least one observer to have at least one swollen joint at the time of locomotion scoring.

No animals in either group were identified as having hair loss over joints. No animals were identified as being lame or having swollen joints prior to individual chute evaluation. Most calves identified with swollen joints had normal or mild locomotion scores. While both heifers identified as lame (Score 2) on locomotion scores were culled early due to lameness, the steers were not.

At the end of the trial, when the mats were to be removed, in those calves with a noticeable amount of manure accumulating, manure was removed from the tails of those calves to prevent discomfort, sores, and tail necrosis. Although the bodies and limbs of some of the calves were dirty, accumulation or matting was not severe, so grooming of the whole animal did not occur. Information about the severity of accumulation and location on the tail, switch vs. body of the tail, was recorded (Table 2). A mild score indicated that the accumulation was removed with minimal effort, either by gloved hand or with scissors. A moderate score indicated that significant time in the chute and/or additional tools were needed to remove the accumulation. A severe score was recorded if any skin damage was noted on the tail.

Within 60 days of mat removal, 10 heifers calves (30%) and 4 steer calves (15%) with mat access were culled from the slatted barns and sold due to lameness. These animals were all sold at salebarns, so it is unknown if they were purchased for slaughter or returned to other farms for additional feeding or possibly breeding (in the case of the heifers). Time on feed for these calves ranged from 146 days to 184 days. The average time on feed for these animals was 163 ± 15 days, with a median of 153 days.

No other health issues besides lameness were noted in these groups. Animals identified as lame showed signs of stiffness, reluctance or difficulty rising, and reduced weight bearing on one or more limbs. Shoulders, hocks, and knees (carpi) were identified as the source of the pain in different culled calves. No foot issues were identified. All culling decisions were made by the manager of the farm (JM).

Three additional heifers (10%) and 1 (4%) additional steer from the original 56 animals were culled for lameness prior to when they would have ideally been sent to slaughter. They were also sent to a salebarn, but it is likely that they would have been purchased for slaughter at that point (a condition known as “realized” in the beef industry, where an animal is sold for slaughter at a less-than-ideal weight). In total, 43% of the heifers and 19% of the steers with access to mats were sold due to lameness.

For comparison, there were 69 steer calves that entered the feedlot at the same time as the 56 study calves. These steers were not on our study, but they were from the same early-weaned, fall-born cohort. In total, five out of this group were culled, and one was euthanized, due to lameness (9%). The one animal euthanized for severe lameness was submitted to the diagnostic laboratory for necropsy. Necropsy revealed arthritis and severe chondronecrosis in multiple joints; no *Mycoplasma* was isolated.

## 4. Discussion

Lameness is a significant welfare concern in indoor-housed finishing cattle on fully slatted floors, even with rubber coating on the slats. This initial trial examined the feasibility of adding supplemental mats to improve animal comfort. To the best of our knowledge, this is the first study looking at adding additional mats positioned on top of rubber-coated slatted floors in an indoor feedlot. Elmore et al. [36] looked at a pen design with a solid rubber mat and bare concrete slats. They found that knee and hock swelling in animals housed in this type of pen were similar to those housed upon bare concrete slats alone; however, this may have been due to the exposure of both groups to the concrete. They also used one solid sheet of rubber covering 60% of the pen, whereas this project utilized multiple small mats placed adjacent to each other that covered approximately 20% of the floor space of each pen. There was a risk that these mats may have become dislodged and potentially fallen into the pit. This did not occur.

We selected the Flexgard stall mats due to cost and accessibility. These mats are firm and typically used in horse stalls, but there are other types of materials designed for use in dairy cow stalls that could be explored, if a clear benefit from the solid mats could be demonstrated. Because these mats had not been used before, it was unknown how big of an area to create, as well as how long to leave the mats in the pen. The number of mats placed in each pen allowed the calves to have an equal amount of space along the back of the pen with mats and without mats. The decision to remove the mats after 89 days (approximately 3 months) was based upon our focus on early-weaned calves. This allowed the calves to have access to the mats for the entire time they were on slats as early-weaned animals. The mats were removed at approximately the time that normally weaned fall-born calves would enter a feedlot. From a practical standpoint, the mats stayed in place, and the animals used them. Despite not having enough area covered with the additional mats for all the animals to lie on them simultaneously, there was enough non-supplemented space for the animals to avoid lying on the mats if they chose to. Over time, as the calves became more accustomed to the observer, calves that were lying on or standing on the mats remained on them, while the calves on the non-supplemented slats continued to approach the observer. This recumbency finding may be an indication of improved comfort with the supplemental mats. It is not a true preference test, such as in Lowe et al. (2001) [48], but it can give an indication that the animals preferred to use them. This is consistent with what Elmore et al. reported, that despite issues with cleanliness, finishing steers preferred to rest on solid rubber mats. A different interpretation could be that the animals did not rise for fear of slipping; however, this does not give a good explanation for the animals that were standing on the mats and remained on them.

A major drawback of solid mats is that they retained both solids and liquids and made the animals dirty, a problem slatted floors were designed to mitigate [48]. The decision not to alter the farm’s daily cleaning routines in this study may have exacerbated this issue. In future studies, incorporating regular cleaning protocols, such as scraping or hosing down the mats, would be necessary to maintain a higher standard of animal cleanliness and welfare. Scraping was not implemented, because introducing a person into the pen would likely have impacted animal behavior; however, moving forward, this is the option we would use. There were several reasons hosing of the mats was not used to help keep the mats clean: the weather at that time of the year, the moisture from hosing, and the manure pits. February temperatures in our area of the country are typically between −6 °C and 3 °C, while in March, temperatures range between −1 °C and 10 °C [49]. Although we had a much warmer than normal winter in 2023–2024, we did not anticipate being able to use water to clean the mats without creating an additional slipping hazard and potentially soaking and chilling the calves. Additionally, the pits under our barns are only 0.91 m deep, which is much shallower than many deeper-pitted barns (over 3 m deep) [50]. We did not know how additional water would impact the manure management system.

The unusually warm weather also resulted in the curtains being left open for most of the observation period. This increase in ventilation may have impacted ammonia concentrations in our barn in a positive manner; however, it is more likely that the low ammonia concentrations were the result of low accumulation of manure in the pits during the first few months of occupancy. Because our pits are shallow, ammonia may not be a major health concern in our facility.

The heifer calves were observed to be lying on the mats more often and at greater numbers than the steer calves; this could very well have been due to the cleanliness of the mats, as the heifers tended to be cleaner and their mats drier. This may indicate that the mats we used are more suitable for heifers than for steers. This observation may be simply due to the direction in which the different sexes urinate, with the steers tending to urinate on the mats. A recent technical report describing a variety of housing systems in the EU [11] supports this and describes a cubical housing system with sloped mats to address this issue for steers and bulls.

Winter-hair coats and small body size may also have played a role in cleanliness, as all groups became cleaner as they shed out in the spring and became larger. This agrees with the findings of Murphy et al., 2018 [42], who also found that there was no effect of floor type on the cleanliness of bulls at the end of the growing and finishing periods. As they grew, there was less of an accumulation of manure on the slats. As the animals became larger, they took up more room in the pen and also pushed more of the manure through the slats. Tail cleanliness was recorded, because although the animals became cleaner as they grew larger and shed out their winter hair, the tails continued to collect manure. Tail injury and amputation have been reported as a common injury in slatted floor feedlots [51], and although that is not something that is seen often in our feedlots, we did not want to contribute to the problem.

Average daily gain was calculated for the early feeding period. It was not anticipated that the mats would have a major impact on production. The ADG for these calves was comparable to what is typically seen in our system. This is comparable to what has been reported in the literature; flooring type does not have an appreciable impact on performance measurements like ADG [12,26,35,36].

Current methods of detecting lameness in beef animals in indoor confinement systems are challenging under production conditions, mainly due to population numbers and density. Despite regular observations multiple times per day and inspecting the animals closely at the level of their carpi, lame animals and animals with swollen joints were not detected until they were run individually through a chute. No areas of hair loss were noted on the hocks or knees, but it would have been difficult to detect that on individual animals due to the dirtiness of the limbs on most of the animals.

We were interested in finding out what was occurring in the animals at our facility that were strictly here for production purposes; this is a standard-sized group of animals for a pen, and our groups that are not on research do not often leave the pen. This is typical of commercial production systems. Although there are well-established scoring systems for assessing integument alterations [52], it was not the intent of this project to individually score animals, because that would require that the calves be examined in a chute regularly. One of the goals was to see what occurred in our production animals without adding any additional bouts of exercise.

The most notable of the early observations of these animals was the lack of movement within the pen and the lack of play behavior. Play behaviors in calves have been described as running, head butting, mounting, jumping, chasing other calves, and head-butting objects around them [53]. A few instances of mounting were observed, as were instances of running and jumping. Occasionally when running, an animal would slip, but no falls were seen. The most common activity noted was a couple of animals manipulating chains or gates. Oral manipulations by calves can be difficult to interpret, as they can be a sign of exploratory behavior, which is generally positive, or they can be a sign of a stereotypy, which can be a sign of frustration [54]. Hanging devices, such as chains or ropes, have been used as environmental enrichment for calves as well [55]. While there were chains available that a few of the calves interacted with, their purpose was to hold gates shut, and they were not selected or placed with strategic enrichment in mind. Adding some sort of physical enrichment to the pens could be a way to increase play behavior and movement within the pens [9]. This technique has been shown to increase mental and physical stimulation in young calves.

Experimental studies have looked at dairy cow preference in selecting bedding substrate, stall designs, and walking surfaces [56,57,58], as well as at the time budgets of dairy cows, particularly focusing on lying behavior [33]. While time spent lying and ruminating seems inherently important in a milking cow with high metabolic demands, time spent exercising (walking, running, playing) seems equally important in young growing animals, particularly those putting on a lot of muscle mass. Additionally, methods of detecting lameness, such as locomotion scores, require the animal to be moving to be evaluated. There seems to be a gap in the literature regarding time budgets of beef cattle or even group-housed calves, particularly with respect to activity levels; however, some work in this area has been conducted in young growing calves in Japan [59].

Locomotion scoring was performed at one time during this study, and it did not appear to be a good indicator of which animals would be culled early for lameness. However, gait scoring did illustrate that 30% of both groups of animals were identified as lame within the first 89 days of being housed on slats. Of the nine heifer calves and eight steer calves with abnormal locomotion scores, only three heifer calves (33% of the heifers with an abnormal gait) and two steer calves (25% of the steers with abnormal gaits) identified as lame were sold early. The other animals sold early were not identified as lame at this point in time. There are a couple of reasons this may be the case. The locomotion scoring may have been carried out too early. It is possible that there was not very much joint pathology at the point in time that locomotion scoring occurred. More training on locomotion scoring may be needed. The difference between a score of zero and a score of one is very subtle, and if all the animals were short-strided, it could be easy to mistake a Score 1 for a Score 0. The calves had also been used to walking on slats, and, perhaps, the locomotion scoring would be more relevant if it occurred on slats. It is also possible that animals can have joint pathology and still have a normal locomotion score.

Additionally, although locomotion scoring has been developed for beef cattle [60] and has similar criteria to that in dairy cattle, it may be more difficult to perform, because beef cattle have a more compact body structure than Holsteins and, in the US, tend to be all black [61]. It was also developed for assessments of cattle in outdoor feedlots and has not been validated for animals raised on slats.

Animals ultimately identified as lame showed signs of stiffness, reluctance or difficulty rising, and reduced weight-bearing load on one or more limbs. Shoulders, hocks, and knee were identified variably as the source of the pain. No foot issues were identified, although the feet were not picked up for examination. These findings were consistent with previous clinical observations and the findings of Terrell et al. [25], who identified upper limb lameness and undefined etiologies to be the most prevalent causes of lameness in commercial beef feedlots. Better methods to detect lameness in beef cattle in indoor confinement systems are needed, as well as a better understanding of the pathology of that lameness. This area could be investigated in the future with newer wearable technologies that have the potential to identify lameness earlier [62,63].

Although we do not want to draw conclusions from comparisons with a different group of calves, the outcomes are interesting. All the calves were born from the same herd in the same environment. The calves on this project arrived in February and had high attrition rates due to lameness, 43% in heifers and 19% in steers, while the steer calves on a different but contemporaneous study only had a 9% attrition rate. One difference between the calves that arrived in February was that the 69 steer calves were weighed at least every 28 days (every 14 days initially) from arrival to finishing, while both groups of calves with supplemental mats only left their pens three times. The chute with the scale is in a different building with grooved concrete floors and dirt and gravel walkways and holding pens. The amount of exercise on good footing received on a regular basis by the 69 research steers could be a factor difference in the lower incidence seen in that group of animals. Alternatively, the supplemental mats or the removal of the mats may have negatively impacted the development of the calves in this project. Because of the different variables and as a consequence of not having a control group, it is not possible to prove the influence of the supplemental mats on the lameness outcomes observed in these results.

Age upon entry to indoor slatted feedlots as a risk factor for lameness has not been extensively investigated. Studies focused on flooring in indoor housing frequently report starting weight of animals on the trial and not the age. Weight and age should correlate to a certain degree; however, some studies begin with a certain age and weight range, yet do not report the age and weight of the animals at the beginning of the feeding period [36,39]. Grooms and Kroll [7] reported the incidence of lameness in cattle in feedlots in the US was 1.9% in 1999 and 1.8% in 2011, which suggests that lameness is perhaps both under-recognized and under-reported. More recent work from information collected in outdoor feedlots suggests that these numbers are much higher. A retrospective study by Davis-Unger et al. 2019 [64] out of Alberta, Canada, which has feedlots similar to the large western feedlots in the US, reported a prevalence of lameness ranging from 1.3% to 46%, while Terrell et al. [25] reported that 38% of animals identified as lame died or were euthanized. This supports the argument that our ability to detect lameness before it becomes severe can be greatly improved.

Both Brscic (2015) [38] and Cozzi (2013) [6] and colleagues have described 8 h behavioral observation periods rather than our 30 min observations, something that might be important to consider incorporating in future projects. In this project, we opted for shorter, more frequent observations to ensure that the supplemental mats were not being moved by the cattle. An eight-hour observation period with multiple observers is a well-established method that would make results easier to compare across facilities. It also gives a better picture of animal behaviors and activities throughout the day. Additionally, those same investigators have conducted much of the recent work examining welfare and performance in feedlot animals on slatted floor barns. However, it is important to note that although there are similarities in some of the challenges with these facilities, there are also differences in the beef industry between the Unites States and Europe, so although the facilities are the same, the animals and management factors may be quite different. For example, it would be unusual to feed out bulls in a US feedlot, while it is illegal to administer or feed growth promotants in Europe [65].

Beef cattle in the US typically obtain a finishing weight of 567–635 kg, but those in intensive housing systems reach those weights much quicker (12–14 months) than those that receive backgrounding prior to finishing (16–18 months). Many research studies [35,36,37,66,67,68] have looked at different flooring types from the perspective of external forces creating lameness, such as abrasive surfaces causing lesions or slippery surfaces causing falls and trauma. However, it is important to consider internal factors such as genetics for rapid growth, nutrition, and biomechanical forces on developing joints and long bones.

In our research setting, because our animals are frequently weighed in a chute, it would be possible in the future to palpate joints to monitor for swelling and quantify the time of onset as well as the specific number and location of joints involved. Other diagnostic modalities such as radiography, ultrasound, and joint fluid cytology could give more insight into the pathology. There are several articles in the literature ascribing joint swelling in feedlot animals to *Mycoplasma bovis*, either as a primary cause or as a sequela to respiratory disease [69,70], but we have not isolated that organism on any of our necropsies over the past 10 years.

In this trial, it is unclear if the presence or subsequent removal of extra mats had any impact on the lameness issues seen. Although the heifers’ growth rate was not as high as that of the steers, it was still well above the averages seen in outdoor systems [71]. This growth rate, combined with limited movement, could be putting enough physical stress on the bones and joints to create the lameness observed but does not explain the rate of attrition of the heifers compared to the steers. Interestingly, of the many studies investigating the impact of flooring on beef cattle welfare, few of the study populations included heifers, and those that did [35,67] did not compare them to steers.

Overall, of the 26 steer calves that arrived in February, 5 were sold early due to lameness, and the rest reached finishing weight. The average days on slats for all steers (finished and early culls) was 257.3 ± 44.1 days. When evaluating the performance of the steer calves without the inclusion of the lame animals, the average number of days on slats was 276.0 ± 8.9 days. Of the 30 heifer calves that began the trial, 13 were sold early due to lameness, and the rest reached finishing weight. The average days on slats for all heifers was 241.3 ± 58.6; when the lame animals were removed, the average time spent on slatted floor was 283.4 ± 21.7 days. The average number of days on slats for heifers removed for lameness was 186 ± 43.2 days on slats, while for steers it was 179 ± 47.5 days. The older animals culled closer to a normal finishing weight skew the average and impact the standard deviation. Furthermore, of the animals that did reach the targeted finishing weight, there was no evaluation of lameness on them, so they could have been both finished and lame.

This study did not collect data on individual mat usage or its correlation with early culling, which would be valuable for understanding the animals’ preferences and welfare outcomes. Future research should include preference testing and accurate time budget assessments to better understand the welfare implications of flooring modifications. Additionally, the outcomes of this study suggest that sex differences may exist in response to modifications.

## 5. Conclusions

This project demonstrated that adding supplemental mats to fully rubber-slatted barns is feasible, but additional maintenance is required to keep the animals clean. The mats contributed to an increased manure accumulation and dirtiness of the animals. Although the calves used the mats for both lying and standing, we are unable to draw conclusions on the impact of the supplemental mats on their welfare. However, based upon the high culling rate of these calves due to lameness, it can be concluded that their overall welfare during the feeding period in indoor housing was not good. While we did not have a contemporaneous negative control group, comparison of the calves with mats with those in a separate project demonstrate higher rates of lameness and early culling in the calves with mats, particularly in the heifer group. Despite providing above-average floor space allowances, heifer calf attrition was high.

Frequent observations of the calves while in their pens failed to reveal useful clinical signs or behaviors to help identify early signs of lameness. This project illustrated the need to develop better methods for detecting and preventing lameness in these systems. Calves brought into indoor slatted floor feedlots at an early age may have additional needs in order to cope well with their environment. In addition to investigating more comfortable lying areas, environmental enrichment that stimulates activity or turnout of some form may benefit this group of animals by providing exercise, as well as allowing observation of meaningful behaviors to detect lameness. The role of exercise and sex-based factors on joint development in these systems should also be evaluated.

It does appear that we were able to achieve a more comfortable lying area. To benefit from this, letting calves out of the pens in order to scrape the mats would provide an opportunity for the calves to get exercise on solid footing, and for the producer or farm manger to have the opportunity to assess lameness in young calves while they are moving. Doing this on a regular basis would keep the pens and animals clean while potentially preventing and/or identifying lameness at earlier stages. This additional cost of maintenance could be well worth the time invested to prevent losing animals.

## Figures and Tables

**Table 1:** Locomotion scoring (LS) agreement between scorers. Individual animal counts and, in parentheses, percentage of the group.

Heifers					Steers				
	Scorer 2					Scorer 2			
Scorer 1	LS 0	LS 1	LS 2	LS 3	Scorer 1	LS 0	LS 1	LS 2	LS 3
**LS 0**	21 (70%)	0	0	0	LS 0	18 (69%)	0	0	0
**LS 1**	1 (3%)	6 (20%)	0	0	LS 1	3 (12%)	3 (12%)	0	0
**LS 2**	0	0	2 (7%)	0	LS 2	0	1 (4%)	1 (4%)	0
**LS 3**	0	0	0	0	LS 3	0	0	0	0

**Table 2:** Number of animals with buildup of manure on tail. Percentages are given in parentheses.

Heifers		Steers	
Dirtiness of tail			
Insignificant	15 (50%)	Insignificant	7 (27%)
Mild	11 (37%)	Mild	10 (38%)
Moderate	4 (13%)	Moderate	9 (35%)
Severe	0	Severe	0
Location			
Switch	12 (80%)	Switch	13 (68%)
Body of tail	3 (20%)	Body of tail	6 (32%)

## Data Availability

The data presented in this study are available within this article or upon request from the corresponding author.
